# Excess congenital non-synonymous variation in leukemia-associated genes in *MLL−* infant leukemia: a Children’s Oncology Group report

**DOI:** 10.1038/leu.2013.367

**Published:** 2014-01-10

**Authors:** M C Valentine, A M Linabery, S Chasnoff, A E O Hughes, C Mallaney, N Sanchez, J Giacalone, N A Heerema, J M Hilden, L G Spector, J A Ross, T E Druley

**Affiliations:** 1 Department of Genetics, Washington University School of Medicine, St Louis, MO, USA; 2 Department of Pediatrics, Washington University School of Medicine, St Louis, MO, USA; 3 Department of Pediatrics, University of Minnesota, Minneapolis, MN, USA; 4 Department of Pathology, Ohio State University, Columbus, OH, USA; 5 Department of Oncology/Hematology, Peyton Manning Children’s Hospital at St Vincent, Indianapolis, IN, USA; 6 Masonic Cancer Center, University of Minnesota, Minneapolis, MN, USA

**Keywords:** infant, exome, *MLL3*

## Abstract

Infant leukemia (IL) is a rare sporadic cancer with a grim prognosis. Although most cases are accompanied by *MLL* rearrangements and harbor very few somatic mutations, less is known about the genetics of the cases without *MLL* translocations. We performed the largest exome-sequencing study to date on matched non-cancer DNA from pairs of mothers and IL patients to characterize congenital variation that may contribute to early leukemogenesis. Using the COSMIC database to define acute leukemia-associated candidate genes, we find a significant enrichment of rare, potentially functional congenital variation in IL patients compared with randomly selected genes within the same patients and unaffected pediatric controls. IL acute myeloid leukemia (AML) patients had more overall variation than IL acute lymphocytic leukemia (ALL) patients, but less of that variation was inherited from mothers. Of our candidate genes, we found that *MLL3* was a compound heterozygote in every infant who developed AML and 50% of infants who developed ALL. These data suggest a model by which known genetic mechanisms for leukemogenesis could be disrupted without an abundance of somatic mutation or chromosomal rearrangements. This model would be consistent with existing models for the establishment of leukemia clones *in utero* and the high rate of IL concordance in monozygotic twins.

## Introduction

Infant leukemia (IL), defined as leukemia within the first year of life, is an extremely rare, sporadic and often fatal cancer. Unlike leukemia in older children where survival rates for acute myeloid leukemia (AML) and acute lymphocytic leukemia (ALL) are approximately 60% and 85%, respectively, infants with leukemia have a 5-year event-free survival rate of about 50%.^1^ Unfortunately, despite years of research and clinical trials, overall survival for IL has not improved substantially since the advent of hematopoietic stem cell transplantation; infants that survive are often left with lifelong deficits in cognition, development, end-organ function, behavioral milestones or other complications because of treatment intensity.^2, 3^

Although other translocations (for example, *ETV6-RUNX1, E2A-PBX1*) commonly seen in pediatric leukemia are very rarely observed in IL, *MLL* rearrangements (*MLL*+) are observed in approximately 50–80% of infant ALL cases and 34–50% of infant AML cases.^4^ In contrast, in older children the percentages of *MLL* rearrangements are 6% and 14% for ALL and AML, respectively.^5^ There is evidence from multiple *in vitro* systems that the presence of a *MLL* rearrangement is insufficient by itself to drive leukemogenesis,^6, 7, 8^ suggesting that additional factors are required in the presence (and absence) of *MLL* rearrangements to drive leukemogenesis. In contrast, very little is known about the role of congenital variation in *MLL−* IL. Infants without *MLL* rearrangements have superior outcomes^9^ and a unique expression profile compared with *MLL+* IL and childhood B-precursor ALL.^10^ Finally, exome^11^ and genome sequencing^12^ of *MLL+* cases have identified exceedingly few somatic mutations, supporting the hypothesis that somatic mutation alone is not the sole driver for IL. In comparison, no such large-scale sequencing effort has been reported on *MLL*− IL cases.

In addition to genetic variation, epidemiological evidence suggests that maternal exposures during pregnancy, particularly to DNA topoisomerase II inhibitors,^13, 14, 15^ predispose to infant AML in a model similar to therapy-related AML after epipodophyllotoxin-containing therapy. Moreover, the number of IL cases increased from 1992 to 2004, consistent with an environmental modifier.^16^ However, the number of compounds that function as DNA topoisomerase II inhibitors are many and exposures are extensive across the population at large, yet IL remains a very rare disease. These observations led to the creation of the Children’s Oncology Group (COG) AE24: ‘Epidemiology of Infant Leukemia’ case–control study to acquire information and DNA samples from mothers and their infants with acute leukemia. The AE24 study observed a statistically significant association with topoisomerase II inhibitor exposure during pregnancy only in mothers of infants with *MLL*+ AML, but this association was not observed for infants with *MLL*− disease or ALL.^13, 17^

In summary, IL is a distinct clinical entity with evidence for both genetic and environmental causes, but detailed studies have failed to account for overall disease incidence. Recently, large-scale genome sequencing projects have revealed that the spectrum of genetic variation in human populations is dominated by rare alleles,^18^ and that these rare variants have greater effect sizes^19^ and are more likely to act dominantly.^20^ Accordingly, some have proposed that rare, recently derived variants segregating in families may be critical for complex disease—and that the phenotypic consequences of these alleles may be critically shaped, and thus variable, by their genomic context (interactions with additional variants).^21^ Motivated by this so-called ‘clan genomics’ framework and the genetic architecture for carcinogenesis proposed by Vogelstein (2–8 ‘driver’ mutations in genes regulating cell fate, cell survival and/or genome maintenance per cancer),^22^ we set out to test whether or not IL arises within a genetic background enriched for functional variation in leukemia candidate genes—potentially mitigating the need for the expected burden of somatic mutation, but still resulting in the expected total amount of variation typically observed in cancer. We have completed exome sequencing of non-cancer DNA from mothers and infants with *MLL*− IL to characterize profiles of rare, congenital germline variation that may influence leukemogenesis and proliferation. Supporting our hypothesis, we observed a statistically significant enrichment of rare, non-synonymous and predicted functional sequence variants in genes previously known to harbor functional somatic mutations in pediatric and adult leukemia. Despite being uncommon, the study of the genetics driving rare cancers and their accompanying predisposition syndromes (for example, retinoblastoma, pleuropulmonary blastoma, hereditary non-polyposis colorectal cancer) has historically elucidated important biological insights into normal and pathological cellular mechanisms that can extend our understanding of additional complex diseases.

## Materials and methods

### Patient information and DNA samples

DNA samples and demographic and clinical information were collected from 23 pairs of deidentified Caucasian mothers and their infants with acute leukemia without *MLL* gene rearrangements who were enrolled on the COG-AE24: ‘Epidemiology of Infant Leukemia’ protocol. Briefly, infants (<12 months) with a confirmed diagnosis of ALL or AML during the period 1996–2006 at North American COG institutions were eligible for the parent AE24 study; cases with Down syndrome were excluded. None of the infants included in this study were reported to have birthmarks, birth defects, known chromosomal abnormalities or family histories of pediatric cancers. In addition to providing buccal cell samples for themselves (via mouthwash) and their infants in first remission (via cytobrushings) using Puregene Buccal Cell Kit (Gentra Systems, Minneapolis, MN USA), as well as consent for genetic research using the samples, mothers also released their child’s diagnostic information, including results of Southern blot, reverse transcription-PCR, fluorescent *in situ* hybridization or other cytogenetics testing, to permit central review. Three independent reviewers evaluated submitted materials to confirm diagnoses and determine if there was evidence of *MLL* gene rearrangement (*MLL+*), no rearrangement (*MLL−*) or insufficient evidence to classify. Institutional Review Boards at the University of Minnesota Coordinating Center (#0309M52104) and participating COG institutions approved the parent AE24 study. Control pediatric exomes were obtained from Caucasian infants and their parents without cancer collected as part of an exome sequencing initiative conducted by the Newborn Medicine Division at St Louis Children’s Hospital (courtesy of F Sessions Cole, MD). Exome sequencing was approved by Washington University Human Research Protection Office ID# 201105062.

### Exome sequencing and data analysis

For all samples, 15–25 ng of germline DNA was whole-genome amplified using the Sigma GenomePlex kit according to the manufacturer’s protocol (Sigma, St Louis, MO, USA). From each amplified product, 1 μg was used for sequencing library preparation according to the Illumina TruSeq DNA Sample Prep v2 kit followed by hybridization capture of each exome according to the Illumina TruSeq Exome Enrichment Kit (Illumina, San Diego, CA, USA). Libraries were sequenced three/lane on the Illumina HiSeq 2000 platform generating 101 bp paired-end reads by the Genome Technology Access Center at Washington University.

For all exome data from probands, mothers and controls, we used a published bioinformatic pipeline^23^ with sensitivity of 96.9% and specificity of 99.8% with exome analysis for raw data alignment and variant calling. Raw sequence data in fastq format were aligned to the NCBI human genome build 37 (hg19) using a purchased, multi-threading version of Novoalign version 2.05 (www.novocraft.com) and published thresholds. An alignment threshold of 200 was used (-t 200), with adapter stripping (5′-a AGATCGGAAGAGCG-3′) and quality calibration enabled (-k). Reads with multiple alignments were discarded (-r none, -e 1) and output was in SAM format (-o SAM). Variant calling from the aligned output for the individual exomes was then performed using SAMtools.^24^ The aligned data were converted to BAM format to allow the removal of duplicate reads using Picard ‘MarkDuplicates’. Variants were then called with the SAMtools version 0.1.18 mpileup command, using options -AB –ugf and bcftools ‘view’ with settings -bvcg. Finally, variants were filtered with vcfutils ‘varFilter’ using default settings except retaining all variants with under 99999 reads. This process ultimately yielded a comprehensive list of exomic variants for each subject, including single-nucleotide variants and short insertions and deletions. ‘Raw’ variant calls from each sample were further filtered by retaining only variants with ⩾5-fold coverage/allele (>10-fold/base position), a genotype quality score of ⩾10 and a mapping quality score of ⩾60. Although ⩾5-fold coverage/allele was a bare minimum, it should be noted that our average coverage per variant per exome was 21.5-fold/allele (43-fold/base position). Each of the individual quality score thresholds will only retain a variant position with at least a 95% likelihood of being a true variant. When applied together, the probability of a variant miscall is significantly reduced. All remaining variants were used as input for the ‘variants_reduction.pl’ tool provided with the ANNOVAR software package (http://www.openbioinformatics.org/annovar/).^25^ To enrich for high-confidence variants likely to confer a functional consequence, successive filters were applied, keeping only variants which were non-synonymous and coding or at splice junctions, and were rare (present at <1% minor allele frequency) in either the 1000 Genomes Project (April 2012 release) or in the dbSNP130 Non-Flagged variants lists. Sequencing results are available at the NCBI Short Read Archive under accession number SRP024273.

### Candidate gene selection

Using version 63 (ALL) or version 64 (AML) of the COSMIC database (http://cancer.sanger.ac.uk/cancergenome/projects/cosmic/),^26^ we compiled lists of genes relevant in AML and ALL. To do this, we used the Tissue search feature, selecting samples from ‘hematopoietic and lymphoid’ tissue followed by ‘NS’ for Subtissue type. For AML, we further refined our gene list by selecting ‘hematopoietic’ from the Histology menu and, from the subHistology menu, ‘Acute myeloid leukaemia’, ‘Acute myeloid leukaemia associated with MDS’, ‘Acute myeloid leukaemia myelodysplastic syndrome therapy related NOS’ and ‘Acute myeloid leukaemia therapy related’. For ALL, we selected ‘Lymphoid Neoplasm’ from the Histology menu followed by ‘Acute lymphoblastic leukaemia’ and ‘Acute lymphoblastic B cell leukaemia’ from the subHistology menu. Having filtered by tissue and histology, we selected all genes with sequence variation in our cohort, which yielded a list of 126 ALL-associated genes and 655 AML-associated genes. Thirty-four genes were shared between candidate gene sets. These genes are listed in Supplementary Table 1.

### Hypergeometric and permutation testing

Hypergeometric (Fisher’s Exact) testing was performed using the ‘phyper’ function in the R software statistics package (version 2.15.3; available at http://www.r-project.org). *P*-values (using an *α*=0.005 to increase stringency) were generated by comparing the aggregate number of rare, non-synonymous, predicted deleterious variation in each patient group against either the matched mothers, the unaffected control population, the opposite patient group or the opposite group of mothers. Unaffected controls consisted of 12 unaffected Caucasian pediatric exomes.

Permutation analysis was executed in the R software package using the ‘sample’ function in the base package. Using this function, a distribution of the number of rare (not listed in dbSNP 135 or the 1000 Genomes Project), non-synonymous and predicted deleterious variants (per multiple prediction algorithms using Annovar)^25^ was created by performing 100 000 iterations of randomly selecting the number of genes identified via filtering (126 ALL-associated genes or 655 AML-associated genes).

### Dideoxy sequencing

Confirmatory dideoxy sequencing was performed at Washington University’s Protein and Nucleic Acid Chemistry Laboratory using primers listed in Supplementary Table 2.

## Results

Table 1 shows the maternal and infant demographics in the ALL and AML subgroups. The infants with AML presented somewhat earlier than those with ALL (5.3 months vs 8.3 months), but otherwise there were no differences between the subgroups. Maternal age also did not associate with phenotype.

Table 2 shows that the average amount of congenital coding variation is higher in affected infants than in mothers or unaffected controls. For infants with ALL, our range of 463–3209 (including insertions and deletions) is consistent with the range of 791–1462 single-nucleotide variants per child reported by Chang.^11^ To focus on variants more likely to impart a functional effect associated with acute leukemia, we identified 126 ALL-associated genes and 655 AML-associated genes within the COSMIC database. From these candidate genes, we tabulated the number of congenital variants that were rare, non-synonymous and predicted deleterious. We found an average of 12 variants per ALL patient in the 126 ALL-associated genes and 163 variants per AML patient in the 655 AML-associated genes, both values exceeded the averages of 6 and 132 observed in ALL and AML mothers, respectively, as well as the 2 and 28 observed in controls (Table 3). Rare, non-synonymous variants in infants with ALL or AML were 2.0 and 1.4 times more likely, respectively, to be found in leukemia-associated genes compared with controls (Supplementary Table 3). There was no correlation between the number of rare, non-synonymous and putatively deleterious variants identified and the size of the gene: *R*^2^=0.21 (ALL) and 0.15 (AML). Given the unexpectedly large numbers of variants identified in candidate genes, infants were tested for an enrichment of variation in candidate genes using a hypergeometric test (Table 3). We found that, compared with controls, IL patients and mothers demonstrated a statistically significant enrichment of variation within either set of candidate genes. These results suggest that IL patients are indeed enriched for rare, deleterious variation in leukemia-associated genes.

**Figure 1:**
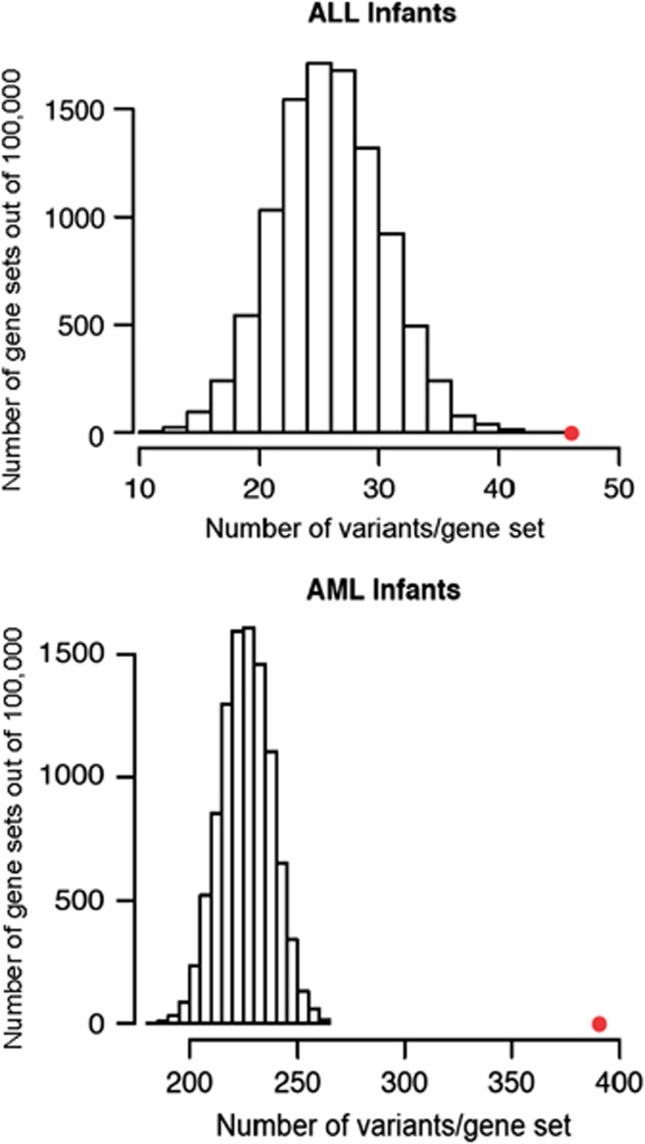
Random permutation testing of gene subgroups in IL ALL and AML patients. The distribution of rare, non-synonymous, deleterious variants in each figure was generated by randomly selecting either 126 (ALL infants) or 655 (AML infants) genes from the patient exomes. The red dot in each panel marks the actual variation observed in each patient group (ALL *P*=3.6 e^−5^; AML *P*=1.0 e^−38^) from each COSMIC candidate gene set.

In order to better address potential biases in the distribution of variation (for example, differing transcript sizes or systematic sequencing error), we performed a randomization test wherein the same total number of genes that were variant in our samples (7808 in ALL and 8422 in AML) was selected at random from each subgroup of IL patients and the number of genes per random sampling that were found on the COSMIC candidate gene lists was recorded (Figure 1). We repeated this procedure 100 000 times and found that observations more extreme than ours were not observed in permutations for either ALL or AML infants, supporting the conclusion that the observed enrichment was not due to systematic errors and was specific to our patients. Alternatively, we also generated 100 000 random lists of only 126 or 655 genes and recorded the number of variant genes observed in each iteration. Results (not shown) were qualitatively the same as our initial permutation experiment. Results of maternal random permutation testing are shown in Supplementary Figure 1. Maternal exomes also demonstrate, to a lesser degree than infants, an enrichment of rare, deleterious variation in leukemia-associated genes but none of these mothers had developed leukemia at the time of study enrollment. We also validated our sequencing variant calls that were unique to an individual with additional dideoxy sequencing (Supplementary Table 2). We did not validate variants observed in matched mothers and infants, as such a result by chance would be exceptionally unlikely.

**Figure 2:**
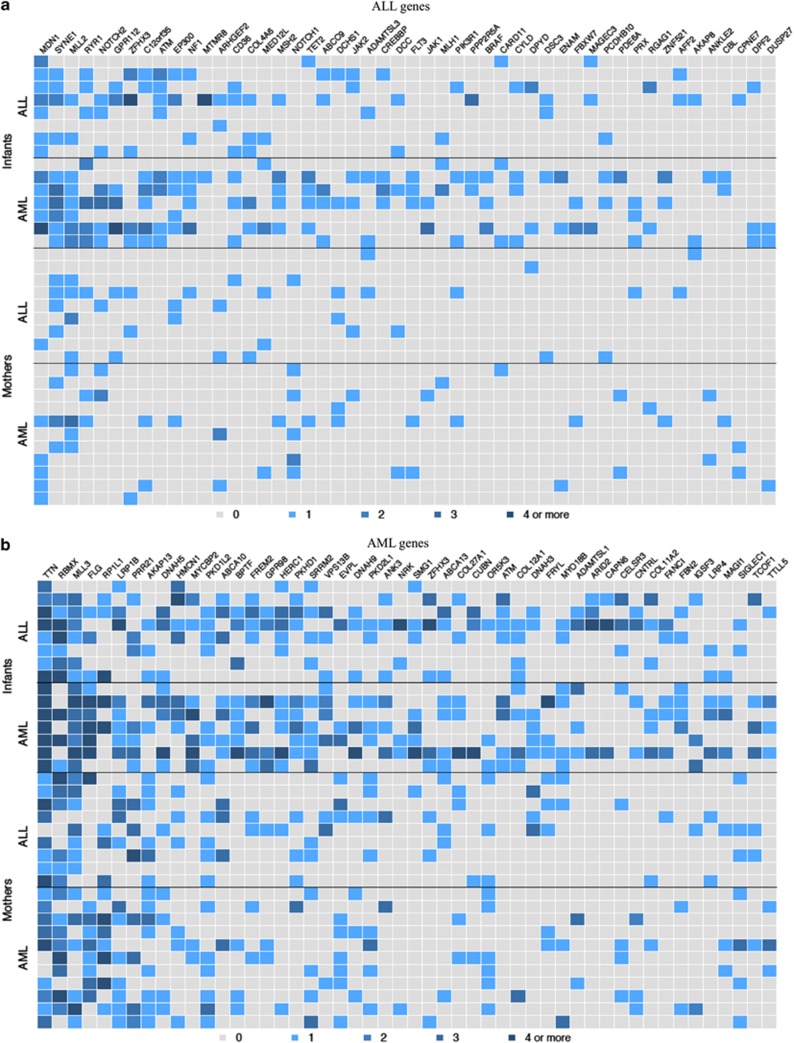
The top 50 variant ALL (**a**) and AML genes (**b**) in infants and mothers. Each row indicates an individual and the row position indicates matched pairs (for example, the top row for ALL infants is the child matched to the mother in the top row of ALL mothers). A colored square indicates a rare, non-synonymous, deleterious variant in that individual in that gene. The shading for each box indicates the number of variants according to the key under the images.

To prioritize genes that may be most relevant to IL and highlight the combinatorial nature of maternal and non-maternal germline variation, we looked for (a) compound heterozygous genes and (b) genes that were the most commonly variant across all patients. We surveyed *all* genes for compound heterozygotes, where a gene must show ⩾2 rare, non-synonymous and deleterious variants with ⩾1 seen in the matching mother and ⩾1 variant that was non-maternal. We found that every AML infant was a compound heterozygote for only two genes: *MLL3* and *ANKRD36*. *ANKRD36* (ankyrin repeat domain 36) was not a leukemia candidate gene and has not been previously associated to leukemia, albeit multiple carcinomas with somatic mutations have been identified in COSMIC. We focused on *MLL3* because it was in our AML-candidate gene list and owing to its direct connection to leukemia. Interestingly, despite the fact that *MLL3* was not on our ALL-candidate gene list, we found that 50% of infants with ALL were also compound heterozygotes. Within *MLL3*, we identified nine stop gain variants (Supplementary Figure 2). Six of these were caused by a known, rare T insertion at chr7:151945072 (rs150073007) and three of these were seen in the matching mothers (four of nine total). For other candidate genes, 67% of AML patients were also compound heterozygotes for *RYR1* and *FLG*, whereas 50% of ALL patients were compound heterozygotes for *RBMX*. We next plotted the top 50 variant candidate genes for infants (Figure 2). We found the most variant (but not necessarily compound heterozygotes) AML-associated genes in infants with AML were *TTN*, *MLL3* and *FLG* (Figure 2b, columns 1,3,4; Infants: AML), but from the ALL-associated gene list, *MDN1*, *SYNE1* and *MLL2* (Figure 2a, columns 1,2,3; Infants: AML) were frequently variable. For infants with ALL, we found that *MDN1* was the most variable ALL-associated gene (Figure 2a, column 1; Infants: ALL), but also noted frequent variation in *TTN*, *RBMX* and *MLL3* from the AML candidate list (Figure 2b, columns 1,2,3; Infants: ALL). Individual variants and their observed frequencies for the top three most frequently variant ALL and AML-candidate genes are listed in Supplementary Tables 4 and 5. Consistent with the hypothesis of a combinatorial inheritance of functionally significant variation in leukemia-associated genes, infants generally show greater variability than mothers. We also observed that infants with AML tend to have more variants across the top genes than ALL infants.

## Discussion

Clearly, a critical component of IL etiology remains undiscovered. The search for these additional insults has been ongoing for decades and has mainly focused on the acquisition of additional somatic mutations within the pre-leukemic clones due either to (a) enhanced mechanisms of mutagenesis from the initial genetic defect or (b) environmental exposures to toxins that promote DNA mutation. Assuming that the typical cancer requires 2–8 mutation in genes regulating cell fate, cell survival and/or genome maintenance,^22^ neither of these mechanisms appears sufficient to account for the incidence of IL, and genome-sequencing results from *MLL*+ infant ALL reported exceptionally few somatic mutations in these three classes of genes.^12^ One category of genetic variation that has not been deeply explored in these patients is rare germline variants. The aptly named model of ‘clan genomics’ by Lupski and colleagues^21^ posits that combinations of rare and private alleles, in the right genomic context, can combine to exert profound, but variable, influence on complex phenotypes. Considering this model, we hypothesized that profiles of rare, coding germline variation may comprise some proportion of the expected functional variation typically observed in cancer, but as of yet, not observed in IL under a model of somatic mutation. Under this model, each parent possesses a partial profile of variation, individually insufficient to significantly increase cancer risk, but through random segregation these alleles align in an offspring and result in the right context to significantly increase that child’s risk of early leukemogenesis. A recent genome-wide association study found support for an additive model of common variants influencing standard-risk pediatric ALL and proposed that additional low-risk and very rare variants are likely to be present and exerting substantial effects on ALL risk.^27^ The Rare Variant Hypothesis posits that a singular complex phenotype may demonstrate a wide variety of functionally significant genetic variants in critical genes or metabolic pathways.^28^ Support for this hypothesis is provided by recent studies asserting that genetic variance for complex traits is mostly additive in nature.^29^ These congenital profiles of variation may consist of very rare variants of strong effect that may be augmented or modulated by additional low-risk variants, which is consistent with reports describing how multiple functional variants are required for a normal cell to undergo malignant transformation.^30^

Although *MLL+* IL has been intensively studied, there are few studies of *MLL−* IL and none simultaneously characterizing large-scale maternal sequencing. To our knowledge, this is the largest sequencing survey of *MLL−* IL. In exome sequencing of non-cancer DNA from matched mothers and their infants who developed acute leukemia, we find a statistically significant excess of rare, non-synonymous and predicted deleterious sequence variants in genes already known to be mutated in hematologic malignancies in the COSMIC database. In addition, mothers demonstrated enrichment in candidate gene variation over the control population supporting the interpretation that the observed enrichment in infants is a chance occurrence resulting from the independent segregation of multiple rare variants inherited from each parent, who individually possess a lesser enrichment of variation in the genes in question. Therefore, consistent with existing models of carcinogenesis,^22, 29^ leukemia can only arise after a discrete threshold of deleterious functional variation is surpassed, whether inherited or acquired. Because paternal, sibling and patient leukemia DNA were unavailable in the AE24 study, our ability to distinguish inherited variation versus *de novo* mutation, identify potentially more penetrant combinations of inherited variants and correlate these patterns with the presence of any somatic mutation is limited. However, the patients in our survey were part of an epidemiologic study that failed to identify significant *in utero* exposures accounting for their IL.^17^ A recent review of *de novo* mutation rates in autistic spectrum disorders reports that only one *de novo* mutation per exome is observed in cases that are significantly enriched for *de novo* mutations.^31^ Thus, although we cannot distinguish paternal variation from *de novo* mutations, elevated rates of *de novo* mutation are insufficient to account for the overall enrichment of variation in candidate genes we have identified in this survey. Despite these limitations, our results continue to support our hypothesis that these infants possess germline variability in leukemia-associated genes and pathways that may reduce the amount of functional somatic mutation typically observed in other cancers.

Given the large number of variants observed, particularly in AML patients, we are not suggesting that every variant identified is involved in leukemogenesis nor that acquired chromosomal rearrangements or somatic mutations are irrelevant. We are providing evidence that these infants harbor an abundance of congenital and putatively functional variation that may drive or modulate early leukemogenesis. Figure 2 qualitatively depicts that approximately three to five genes are commonly variant in the germline of most or all of these infants, consistent with the functional classes of genes and number (two to eight) of variants, thought necessary for carcinogenesis.^22^ This model of germline variation influencing leukemogenesis could explain the short latency that has proven difficult to replicate in animal models given an entirely different profile of background variation. The model would not exclude potential leukemogenic effects of topoisomerase II inhibitor exposure, although this has only been associated with *MLL+* IL, and would also be consistent with the lack of heritability observed within pedigrees if cosegregation of many alleles were necessary to predispose to malignancy. A recent Brazilian study of leukemia in children younger than 2 years found a statistically significant increase in adjusted odds ratios of 1.66 for infants with ALL and a near-significant increase of 1.54 for AML when any second-degree relative had cancer, supporting the conclusion of more subtle familial genetic susceptibility in IL.^32^ Interestingly, the odds ratios increased significantly when the children’s father had any cancer (1.80 ALL and 2.34 AML), but no such significant increase was observed when mothers had cancer.

Additive germline variation could also explain the very high concordance observed in monochorionic twins as both would have the same profile of inherited genetic variation, as well as the relative lack of disease concordance between dichorionic twins despite an 8% incidence of shared placental circulation. The ‘intraplacental metastasis hypothesis’^33^ is useful to describe the exceptionally high rate of leukemia concordance for monochorionic twins who share intra-placental anastomoses. The blood-borne nature of these hematologic malignancies would also explain why other pediatric cancer types do not show similarly high concordance in monozygotic, monochorionic twins.^34^ However, the majority of twins are dichorionic, and approximately 8% demonstrate blood group chimerism because of placental fusion allowing blood exchange.^35^ Despite this frequency and multiple reports of twin–twin transfusion syndrome in dichorionic twins,^36, 37, 38, 39, 40, 41^ we found only one report of concordant IL in dichorionic twins because of the leukemia clone passing between infants, not through inter-placental anastomosis, but through the maternal circulation and not resulting in leukemia in the mother, only the other twin.^42^ Although discordant IL in monozygotic twins is rare, Brown and colleagues documented the apparent spontaneous resolution, potentially through an immune-mediated process, of a *MLL-ENL*+ clone from a co-twin of an affected twin.^43^ These observations further support the conclusion that these circulating leukemic clones require additional factors in order to proliferate.

*MLL3*, a homolog of *MLL*, maps to 7q36, which is a chromosomal region often deleted in myeloid leukemias.^44^ Like its *MLL* family members, *MLL3* is a H3K4 histone methyltransferase that regulates gene expression through the FYR and SET domains.^45^ We identified nine rare or novel germline frameshift insertions that introduces a premature stop codon truncating the C-terminal FYR-N, FYR-C and SET domains (Supplementary Figure 2) necessary for proper target gene expression (for example, *HOX*), critical for embryogenesis and development.^46^ *MLL3* has not been previously linked to IL, but has been associated with a variety of solid tumors and did show an enrichment of mutations in adult AML patients^47, 48^ and germline *MLL3* variation was recently reported in a pedigree with adult-onset AML and colorectal cancer.^49^ In addition to *MLL3*, *TTN* was frequently variable in these patients’ germline and has been previously found to carry somatic mutation and germline variation in multiple cancer types.^47, 50^ Although best studied during embyonal cardiac development during mesoderm differentiation between heart and blood, *TTN* has also been shown to be required for proper chromosome packaging and remodeling during cell division.^51^ It is reasonable to hypothesize that aberrant chromatin remodeling, either through dysfunctional *MLL3* alone or in concert with dysfunctional *TTN* during embryonal mesodermal differentiation, may have a crucial role in the etiology of IL in these *MLL*− cases.

These data raise interesting new insights into the genetic architecture of *MLL−* IL. Future work will focus on additional sequencing of nuclear family pedigrees with an affected infant to further refine the candidate genes influencing leukemogenesis, as well as functional analyses in patient-derived myeloid precursors of profiles of additive variation in the context of *MLL3* and *TTN* dysfunction within iPSC-based *in vitro* and *in vivo* model systems.

## Figures and Tables

**Table 1:** Demographic characteristics of the study cohort

	*ALL*	*AML*
*Sex*		
Boys	4	6
Girls	8	7
Average age at diagnosis (months)	8.3 (0.6–11.4)	5.3 (1.6–11.4)
Average maternal age (years)	31.9 (21.3–40.6)	33.4 (25.4–41.8)
No. of mothers >35 years	3	5

Abbreviations: ALL, acute lymphocytic leukemia; AML, acute myeloid leukemia.

**Table 2:** The average and range of filtered variants per exome in each subgroup

	*Average total variants per exome*	*Range*
ALL infants	1264.4	463–3209
ALL mothers	1112.6	985–1267
AML infants	2549.9	519–5545
AML mothers	1225.0	1000–1660
Unaffected controls	582.8	467–719

Abbreviations: ALL, acute lymphocytic leukemia; AML, acute myeloid leukemia. Total exomic variants (single-nucleotide variants and INDELs) were filtered for variants that were rare (not previously included in dbSNP 135 and the 1000 Genomes Project), non-synonymous, with coverage ⩾5-fold, a genotype quality score ⩾10 and a mapping quality score of ⩾60.

**Table 3:** Hypergeometric analysis of variation in leukemia-associated genes determined by comparing the observed amount of rare, non-synonymous and predicted deleterious variation in the 126 (ALL) or 655 (AML) COSMIC-identified candidate genes against the expected amount of similar sequence variation observed by randomly selecting 126 or 655 genes from the same patients

*Group*	*Average variants/exome*	*Range*	P-value*
*ALL genes (*n*=126)*			
ALL infants	12.1	3–33	3.6 e^−5^
ALL mothers	6.4	3–11	1.4 e^−3^
Unaffected controls	1.9	0–4	0.24*
AML infants	22.7	4–37	3.0 e^−9^
AML mothers	8.2	4–16	1.7 e^−9^
			
*AML genes (*n*=655)*			
AML infants	163.4	38–358	1.0 e^−38^
AML mothers	132.5	60–667	5.3 e^−19^
Unaffected controls	27.5	12–37	0.007*
ALL infants	59.4	24–131	5.2 e^−29^
ALL mothers	49.6	40–67	1.5 e^−11^

Abbreviations: ALL, acute lymphocytic leukemia; AML, acute myeloid leukemia. *P*-values generated from hypergeometric (Fisher’s exact) test with *α*=0.005 (* not significant).
