# Supplementary material for: Excess congenital non-synonymous variation in leukemia-associated genes in MLL− infant leukemia: a Children's Oncology Group report
Source: Leukemia. 2014 Jan 10;28(6):1235–41. doi: 10.1038/leu.2013.367 (PMC4045651; doi:10.1038/leu.2013.367)
Supplement: Supplementary Data [file leu2013367x1.pdf]

Supplementary Data for:

Excess congenital non-synonymous variation in leukemia-associated genes in *MLL-* infant leukemia: A Children's Oncology Group Report.

Running title: Excess germline variation in infant leukemia.

Mark C. Valentine,[1] Amy M. Linabery,[2] Sara Chasnoff,[1] Andrew E. O. Hughes,[1] Cates Mallaney,[1] Nicholas Sanchez,[1] Joseph Giacalone,[1] Nyla A. Heerema,[3] Joanne M. Hilden,[4] Logan G. Spector[2,5], Julie A. Ross[2,5*], Todd E. Druley[1,6*,#]

Departments of Genetics[1] and Pediatrics,[6] Washington University School of Medicine, St. Louis, MO; [2]Department of Pediatrics and Masonic Cancer Center,[5] University of Minnesota, Minneapolis, MN; [3]Department of Pathology, The Ohio State University, Columbus, Ohio; [4]Department of Oncology/Hematology, Peyton Manning Children's Hospital at St. Vincent, Indianapolis, IN.

* These authors contributed equally to the study.

# To whom correspondence should be addressed at:

Todd E. Druley, M.D., Ph.D.

Department of Pediatrics

Campus Box 8116

660 South Euclid Avenue

St. Louis, MO 63108

Phone: 314-286-2124

Fax: 314-454-2780

Email: druley_t@wustl.edu

AML Candidate Gene list:

ABCA10	BCOR	CNGA1	DNMT3A	FFAR1	HK1	KLK7	MLIP	NUBP2	PLCZ1	RP1L1	SPATS1	TRPM4	ZNF689
ABCA13	BECN1	CNPPD1	DOCK2	FGL2	HKDC1	KRAS	MLL3	NUMA1	PLIN1	RPL27A	SPTBN5	TSPYL5	ZNF75D
ABCB11	BICD1	CNTLN	DOCK4	FILIP1	HMCN1	KRT1	MORC3	NYX	PLRG1	RPL9	SRCRB4D	TTC39A	ZNF788
ABCD2	BMP5	CNTN5	DOCK9	FKTN	HNRNPUL1	KRT14	MPL	ODZ2	PLXNA3	RSRC2	SRRM1	TTLL10	ZRSR2
ABCG8	BMPER	CNTNAP4	DOK2	FLG	HOOK3	KRT79	MPND	ODZ3	PNPLA7	RUFY1	SRRM2	TTLL2	ZYG11A
ABL1	BMS1	CNTRL	DRD2	FLJ44216	HRCT1	KSR2	MRPL14	OR1C1	POLR2A	RUNX1	SRSF2	TTLL5	
ACCN2	BOC	COL11A2	DROSHA	FLRT2	HTR1A	L1CAM	MSH6	OR1I1	POU6F2	RXFP1	SRSF6	TTN	
ACSS3	BPTF	COL12A1	DSCAM	FLT3	HTR3C	LAMA5	MSR1	OR1J2	PPP1R9A	SAA4	SSX7	TUFT1	
ACTA2	BRAF	COL19A1	DYNC1H1	FNDC1	HTR5A	LAMC1	MT-ND4	OR4C15	PRDM9	SAP130	STAG2	U2AF1	
ADAM11	BRPF1	COL27A1	DYSF	FOXP1	IDH1	LARP4B	MTMR8	OR51L1	PRICKLE3	SCARB1	STC2	U2AF1L4	
ADAM33	BRWD3	COL2A1	E2F8	FREM2	IDH2	LCE1B	MTUS2	OR51S1	PRODH2	SCEL	STK32A	UGCG	
ADAMTSL1	BTBD8	COL7A1	EED	FRMD8	IFIH1	LIMA1	MUC5AC	OR52H1	PRPF40B	SCML2	STK36	UGT1A10	
AFF2	C10orf118	COL9A1	EFTUD1	FRMPD3	IGHMBP2	LIPC	MYBL2	OR5AR1	PRPF8	SCUBE3	STRN	UNC5B	
AGAP1	C10orf2	COMMD5	EGFL8	FRYL	IGSF21	LIX1	MYCBP2	OR5K3	PRR13	SDK2	STT3B	UQCR10	
AGBL1	C10orf76	CP	EIF2D	FRZB	IGSF3	LOC652153	MYEOV	OR6A2	PRR21	SEMA3A	STX3	USP44	
AHCYL1	C1orf168	CPLX4	EIF4B	FZD1	IKZF1	LONRF1	MYH14	OR8A1	PRSS27	SEMA7A	STXBP2	USP9X	
AKAP13	C20orf173	CREBBP	ELANE	G6PC	IL17RD	LPL	MYLK2	OR8H1	PSG3	SEZ6L	SULT1C2	VCAM1	
ALS2CR11	C3orf25	CSF1R	ELF1	GABRG3	IL1R1	LRIG3	MYO18B	OSTalpha	PTCH1	SF3B1	SUPT5H	VCAN	
AMOTANG	C8orf84	CTCF	ELFN2	GABRR1	IMP4	LRP1B	MYO1F	OVGP1	PTEN	SFXN2	SUSD5	VIL1	
ANK3	CACNA1E	CTSG	ELL	GANC	IMPG2	LRP4	MYOC	P2RY2	PTPN11	SH3TC2	SUZ12	VIP	
ANKRD13A	CACNA2D3	CUBN	ELN	GATA1	INS	LRRC2	NAE1	PA2G4	PTPRE	SHANK1	SV2A	VIPR1	
ANKRD26	CADM2	CUL3	EML4	GATA2	INS-IGF2	LRRC37A3	NALCN	PAMR1	PTPRG	SHQ1	SYTL4	VPS13B	
ANPEP	CALB2	CYBB	ENC1	GATAD2B	ITGA8	LRRC37B	NANOS2	PAPPA2	PTPRN	SHROOM2	TACR3	WAC	
AP1G2	CALD1	CYLC1	EPB41L5	GBP4	ITGAD	LRRC40	NAPA	PARD3	PTPRN2	SI	TBC1D4	WDR11	
AP1M1	CAMTA1	CYP1A2	EPHA8	GCAT	ITGAX	LRRC47	NAT8L	PARP2	PTPRT	SIGLEC1	TBX5	WEE1	
APOL6	CAPG	CYP4F8	EPHB1	GDF10	JAK1	LRRN2	NAV1	PARP6	PTX4	SK681	TCEAL3	WLS	
ARC	CAPN6	DAAM2	ETF1	GDF5	JAK2	LRWD1	NCAPH	PCDHA13	PXDNL	SKOR1	TCOF1	WNK4	
ARHGAP39	CAPS	DAGLA	ETV6	GDPD4	JAK3	LTA4H	NCOA7	PCDHA4	RAB17	SLC12A1	TCTE3	WT1	
ARHGAP5	CARD11	DAGLB	EVPL	GDPD5	JAM2	LUZP2	NCR1	PCDHA6	RAB25	SLC15A1	TET2	XIRP1	
ARHGEF2	CASQ1	DAOA	EWSR1	GLE1	JMJD5	MAGEB1	NDST3	PCDHA8	RAB36	SLC17A3	THEG	ZC3H12D	
ARID2	CBL	DAXX	EXOC2	GLE1L	KAT2B	MAGI1	NDUFA13	PCDHB1	RAD21	SLC24A3	TIF1	ZC3H18	
ARSF	CCND3	DAZAP2	EXOC4	GLI1	KCNA3	MAGI2	NEFH	PCDHB10	RAI2	SLC25A11	TKTL1	ZC3H8	
ASAP2	CDC42	DCAF8L1	EZH2	GLRA1	KCNH5	MAP1B	NEMF	PCDHB11	RANBP2	SLC25A12	TM7SF4	ZDHHC11	
ASTE1	CDH18	DCHS1	EZR	GLTPD2	KCNK6	MAP2	NES	PCDHB7	RASSF7	SLC25A20	TMEM132C	ZFHX2	
ASTL	CDH24	DCLK1	FAM123A	GNAI2	KCNQ2	MAP3K15	NF1	PCDHGA2	RB1	SLC26A2	TMEM151B	ZFHX3	
ASXL1	CDH4	DCT	FAM13A	GNB1	KCNQ3	MAPK1	NF2	PCDHGC4	RBKS	SLC30A6	TMEM169	ZMYND8	
ATG16L1	CDHR2	DCTN1	FAM171A1	GPC2	KCNT1	MASP1	NLRP4	PCMTD2	RBM41	SLC37A2	TMEM198	ZNF211	
ATM	CDKN2A	DDHD1	FAM19A4	GPR183	KCNT2	MAX	NLRP8	PDGFRA	RBM46	SLC4A11	TMTC2	ZNF213	
ATP1A2	CEACAM19	DDX1	FAM27L	GPR98	KDM6A	MBLAC1	NMNAT2	PDK4	RBMX	SMAD4	TNKS1BP1	ZNF236	
ATP1B4	CEBPA	DDX60	FAM57B	GPRC6A	KHK	MDFI	NONO	PDLIM7	RCAN2	SMARCB1	TNR	ZNF260	
ATP2A2	CECR2	DENND2A	FAM5C	GRIK2	KIAA0226L	MED14	NOS1	PDS5B	RET	SMC1A	TNS4	ZNF276	
ATP6V1G3	CELSR1	DHX34	FAM69A	GRIN2B	KIAA0240	MEGF11	NOTCH1	PDXDC1	RFC1	SMC3	TOP3B	ZNF324B	
ATP9B	CELSR3	DIS3	FAM83B	GRIP1	KIAA1217	METTL3	NOTCH2NL	PGLYRP2	RGS8	SMG1	TP53	ZNF34	
B4GALNT1	CEP128	DLGAP3	FANCI	GRM8	KIAA1239	MGC99813	NOTCH4	PHF6	RIMS1	SOCS1	TP53I11	ZNF37A	
BAAT	CEP170	DLX3	FBN2	GSTM3	KIAA1244	MGLL	NOX3	PHF8	RIN1	SOHLH1	TP73	ZNF43	
BABAM1	CHIT1	DNAH3	FBXL18	GUCA1A	KIAA1267	MIER3	NPM1	PKD1L2	RIPK4	SON	TPTE2	ZNF462	
BAI1	CHRNA4	DNAH5	FBXL7	GUCA2A	KIDINS220	MIS12	NRAS	PKD1L3	RNASE9	SORCS3	TRAM1L1	ZNF485	
BBS7	CILP2	DNAH9	FBXO11	HERC1	KIF2C	MKRN3	NRK	PKD2L1	RNF111	SOS1	TRIM24	ZNF616	
BCL11B	CLCN6	DNAJA3	FBXO27	HIVEP1	KIT	MKX	NRXN2	PKHD1	ROBO2	SOX5	TRPC1	ZNF677	
BCLAF1	CLVS2	DNM2	FBXO3	HJURP	KLHL29	MLF1IP	NSD1	PLCH2	RP1	SPATA20	TRPC4AP	ZNF687	

ALL Candidate Gene list:

ABCC9	CCND3	DCHS1	GBP6	MAGEC3	NRAS	PTPN11	TM4SF2
ADAMTSL3	CD36	DENND3	GCFC1	MDN1	OFD1	RASGEF1A	TMEM30A
ADCK1	CD74	DHX15	GIMAP5	MED12L	OR51D1	RB1RB1	TNFAIP3
AFF2	CD79B	DPF2	GPR112	MEF2B	OR8H3	RGAG1	TP53
AKAP8	CDC42EP1	DPYD	HLA-DMB	MEGF10	PAX5	RUNX1	TRAF3
ANKLE2	CDKN2A	DSC3	HMGB1	MLH1	PCDHB10	RYR1	TSC22D1
ARHGEF2	CDKN2C	DUSP27	HNF1B	MLL2	PDCD4	SCNN1A	TSPAN7
ATM	CDKN2a(p14)	DUSP9	IDH1	MSH2	PDE6A	SERPINA1	UBE2A
B2M	COL4A6	ENAM	IGSF5	MTMR8	PHOX2A	SERPINA6	WDR88
BCL2L10	CPNE7	EP300	IKZF1	MYD88	PIK3CA	SMARCB1	ZFHX3
BRAF	CREBBP	ETF1	IL7R	MYOM2	PIK3R1	SOCS1	ZNF311
BRSK1	CRLF2	EZH2	JAK1	NCEH1	PMS1	STOML2	ZNF394
C12orf35	CTNNB1	FBXO31	JAK2	NEMF	PPP2R5A	STRADA	ZNF521
CAMTA1	CXorf48	FBXW7	KLF2	NF1	PRDM1	SYNE1	ZWILCH
CARD11	CYLD	FKBP9	KRAS	NOTCH1	PRX	TET2	
CBL	DCC	FLT3	LRP1B	NOTCH2	PTEN	TLL2	

Supplementary Table 1. COSMIC defined candidate genes for AML and ALL.

Gene	Position (hg19)	Subject with Variant	Reference Base	Variant Allele	AA change	Forward primer	Reverse primer
AFF2	chrX:148035259	Infant	C	T	GCA (A) → GTA (V)	cacctgaactgggctgtttt	ctggcaccttcactcacctc
AIF1	chr6:31584079	Infant	G	C	AGG (R) → ACG (T)	ttggtgagaaacgggtgatttgcggg	ttgagcctgtggacaagggtagggat
ALDOA	chr16:30078672	Infant	G	A	GCA (A) → ACA (T)	taccaatatccagcactgaccccgga	tgttctcggtgccaatggactgca
ATXN7L1	chr7:105255128	Infant	C	G	AGG (R) → AGC (S)	atgagggaaagctgtggtgtgggaca	agcaatgccaacccgatgtctcact
COL16A1	chr1:32156158	Infant	G	C	CCA (P) → CGA (R)	cttcacacctggcttcccctgtta	ctcccttcctttgaaacctagaatggct
COPA	chr1:160262988	Infant	T	C	ACT (I) → GTC (V)	ccccttatgggtaggataatttcccttg	cggcagggatgagtggctaatactt
DDX43	chr6:74104671	Infant	G	A	GTT (V) → ATT (I)	tggcacgctactcttacgacgtca	caaaacacagcggcagttcctcgt
EEF1G	chr11:62339051	Infant	C	T	TGG (W) → TGA (stop)	acagacacttcctccatcaactgccc	tttggaggtgagggtgtaggagagga
EFTUD	chr15:82533640	Infant	G	A	GTA (V) → ATA (I)	cccctctttcctcacccttaaccatt	tgggaaaagttggaatggtagctctgg
EXOC3	chr5:465833	Infant	G	C	GGT (G) → CGT (R)	tgaagagggaggttcccagtcaggaa	cgtgcatcccttacctgatgtctgga
FAM196B	chr5:169310673	Infant	G	C	GGG (G) → GCG (A)	actggctgtgagtctcttccttttga	agtgtatggtcctgatccttcccact
GBA2	chr9:35739005	Infant	C	A	GAC (D) → TAC (Y)	ggagccatggttcatcatctgtggga	attccctgcctcccttctgtgtctct
GPR84	chr12:54756704	Infant	G	A	TCA (S) → TTA (L)	tgcaaccattgagccaggtgaggtt	acaggcaagcatccactccaacca
KCNIP4	chr4:20852245	Infant	G	C	CCT (P) → GCT (A)	ctctgggaattgtgtgaaggta	tccgttttcctttgttgctc
KIF11	chr10:94389974	Infant	G	T	CAG (Q) → CAT (H)	actagctagatatcctaccagccagct	tggcagcatcatgaagtttctcctca
LILRA3	chr19:54802548	Infant	G	T	TCC (S) → TAC (Y)	tgtcactgtctgtctctccctccctt	agggaaagtttgtggggaagcctga
NCAPD3	chr11:134062612	Infant	C	T	CTC (L) → TTC (F)	tggaagtcagtcagggaaagagagacca	agatgcgtgcagatccagaaagcct
NCOA2	chr8:71041055	Infant	C	G	CGG (R) → CCG (P)	gtcttagttgatttggctggttctgcac	tccagagccaagcagtagatccaga
OR2V2	chr5:180582262	Infant	GTCT	deletion	Frameshift	ttcttcctcagccagctctccctcat	accttcctcaagccacagtaggggaa
PHKB	chr16:47684830	Infant	C	A	CAG (Q) → AAG (K)	tgaacacagtgagcccttgggaaga	tgctgcaagtgttcaagcatctgact
PIP4K2C	chr12:57992903	Infant	G	A	CGA (R) → CAA (Q)	tccctgggctgtttgtgtatctgct	aaagaacagtcagaacccagcccct
POLR3A	chr10:79773461	Infant	G	T	CTT (L) → ATT (I)	tggcgttgttagttgtggtggtgtt	agtgcccagtattccaggaagccat
PRDM7	chr16:90142285	Infant	G	A	GAA (E) → AAA (K)	ccccattccaatgcacattcagacaga	agtcttcctgctctgaacacccca
PRRC2C	chr1:171509954	Infant	G	C	GTA (V) → CTA (L)	agcaccaattcagccacagtcagtt	ggcctcgacctctgtatccttgatct
PTGES3L	chr17:41123653	Infant	C	T	GAG (E) → AAG (K)	tcctccagagacatgtgtgcagaga	atatgcccacactctcccacacact
RALGAPA2	chr20:20506962	Infant	C	G	CAG (Q) → CAC (H)	cctctgtctggaatactgctggctt	acacagtgaggctagagaagagacagt
RRAD	chr16:66957510	Infant	G	C	CAG (Q) → CAC (H)	ttccctccccgccagttttctttct	tgtgtgttgtgtgttctccaggacgg
SFTPA2	chr10:81317038	Infant	C	T	GGC (G) → AGC (S)	acacactgctcttttccccgacct	tgcagggctccataatgacagtagga
SYNE1	chr6:152469381	Infant	G	A	GAG (L)→GAA (E)	gggatggagtccacactagc	ctagctcccagacgatgagc
SYNE1	chr6:152570334	Infant	C	A	TGC (A)→TGA (stop)	caaacaaaagtgccactgtga	tgagttttcccgttgctttc
SYNE1	chr6:152576794	Infant	C	T	TTC (E)→TTT (F)	aaaagtgctgtggcaacaaa	ggccccatcctgatattttt
SYNE1	chr6:152631566	Mother	G	A	ACG (R)→ACA (T)	gcttacctgccgatgagaga	tgctcacctgtgatgtggtt
SYNE1	chr6:152631653	Infant	C	A	ATC (D)→ATA (I)	gcttacctgccgatgagaga	tgctcacctgtgatgtggtt
SYNE1	chr6:152642398	Mother	G	A	CGC (A)→CAC (H)	tcacacaagaggactgaccta	ccacaaatcaccgacagaac
SYNE1	chr6:152650962	Infant	G	C	TGT (T)→TCT (S)	tcagcgaggctgtgttctaa	acctcaacctgcaggacatc
SYNE1	chr6:152674464	Mother	C	A	CTT (K)→ATT (I)	tgcctacctccaacgtcttc	ctggcacaggcctttacttc
TRPV4	chr12:110230503	Infant	G	C	ACC (T) → AGC (S)	atgtggtgtgtgtgtgactccctcca	tgtcttcccctccagagcctcattgt
TXNIP	chr1:145440753	Infant	G	A	AGT (S) → AAT (N)	tgttaccacagctgtcttgtttctccag	ccacataagaatcctgcccaagaaatgc
UGT3A2	chr5:36039780	Infant	C	G	GAC (D) → CAC (H)	tgggccaatgagaacactgacactt	aggacatgaccacagtgcagctagt
ZHFX3	chr16:72830465	Infant	G	A	TGG (P)→TAG (stop)	ggtgcaattgtaggtgaggtg	tcaagagcacgttcatcagaa
ZHFX3	chr16:72832557	Mother	C	T	TCC (G)→TCT (S)	ctgaccggttgctgattctt	ggtgccatccttgaaacaaa
ZNF362	chr1:33764618	Infant	G	C	GGC (G) → CGC (R)	tccctgttctgaatctcatccctgc	tctggaagattgggaaggtcgtgag
ZNF717	chr3:75788130	Infant	C	T	GGG (G) → GAG (E)	ctctcctgtgtgtgtctgctgatgt	accaacagcaacacatcaactcagga

Supplementary Table 2: Validation of called exome variants via dideoxy sequencing. Fifty rare, single individual variants with ≥5-fold coverage and a quality score ≥20 were selected for secondary dideoxy validation. Of the 50, 43 (86%) were validated and are listed here. Rare variants observed in both mother and child were not chosen for validation as they are unlikely to be false positives.

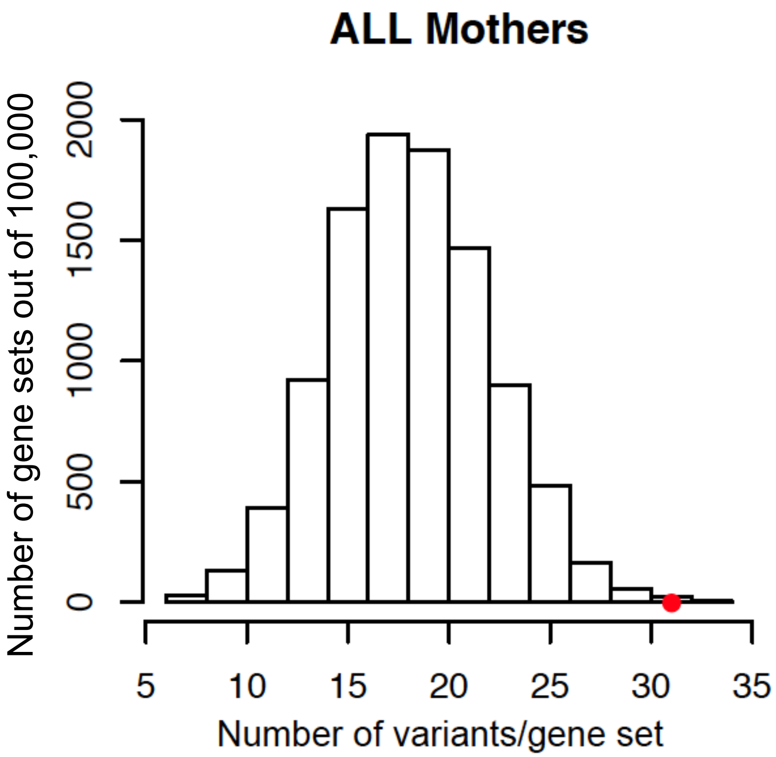

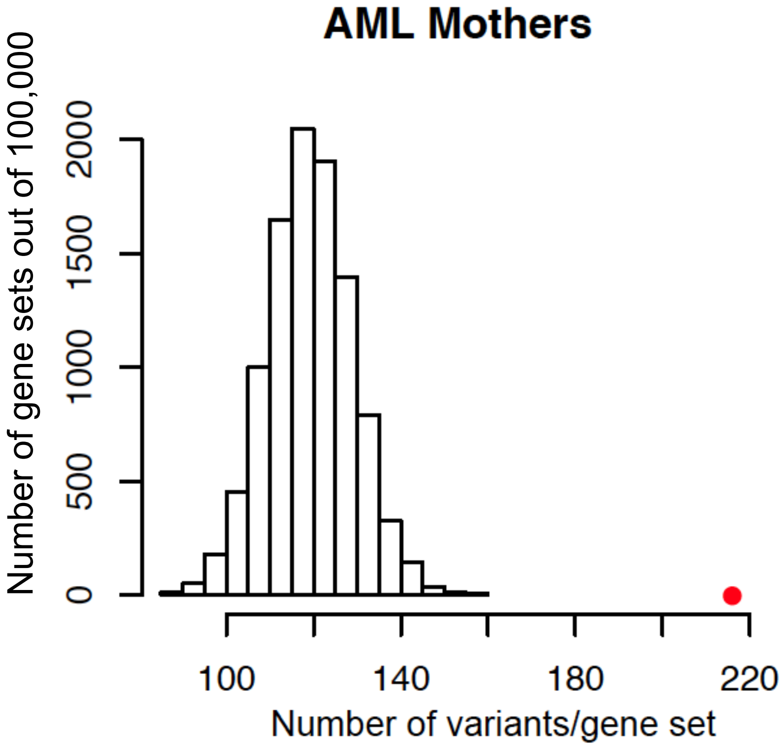

Supplementary Figure 1. Random permutation testing in maternal exomes. The distribution of rare, non-synonymous, deleterious variants in each figure was generated by randomly selecting either 126 (for ALL) or 655 (for AML) sets of genes in 100,000 iterations from the maternal exomes. The red dot (•) in each panel marks the variation observed in each maternal group from each COSMIC candidate gene set. Maternal exomes demonstrate an enrichment of variation in leukemia-associated genes similar, but to a lesser degree, than their infants with acute leukemia.

Leukemia subtype	Subgroup	Likelihood ratio of having a rare, non-synonymous, deleterious variant in a COSMIC-defined candidate gene relative to controls.
ALL	Infants	2.01
	Mothers	1.62
AML	Infants	1.44
	Mothers	1.44

Supplementary Table 3. The likelihood of possessing a rare, non-synonymous and predicted deleterious sequence variant in a COSMIC-defined leukemia-associated candidate gene relative to unaffected controls. Likelihood ratios were calculated as follows:

$$\frac{\text{Number of rare, non-synonymous, deleterious variants (either ALL or AML mothers or infants) / total number of variants in the respective group}}{\text{Number of rare, non-synonymous, deleterious variants in controls / total number of variants in controls}}$$

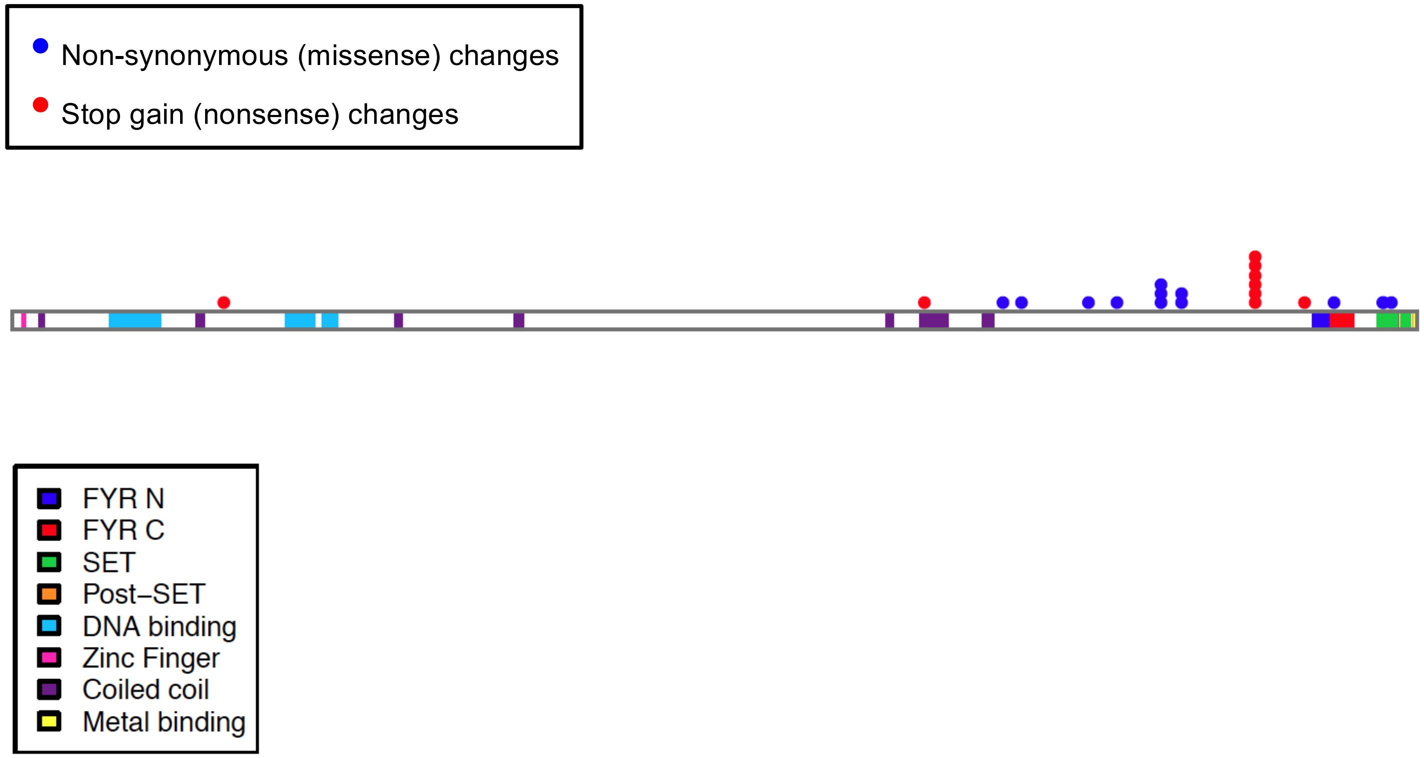

Supplementary Figure 2. Plot of *MLL3* functional domains and positions of germline sequence variation. The bar represents the coding sequence of the *MLL3* open reading frame with color-coded functional domains in their relative positions. The circles above the bar represent base positions where either missense (blue) or nonsense (red) variants were identified by exome sequencing. Nine infants possessed rare nonsense variants (six at the same base position, rs150073007). Four of these nine nonsense variants were also observed in the matching infants' mothers.

Variant list for the top three variant AML-candidate genes (see Figure2, Panel B) - page 1 of 2									
AML Infants					ALL Infants				
Gene	Position	Infant Frequency	Mother Frequency	dbSNP ID	Gene	Position	Infant Frequency	Mother Frequency	dbSNP ID
TTN	chr2:179393490	14%	0%		TTN	chr2:179392343	13%	0%	
TTN	chr2:179395413	14%	0%		TTN	chr2:179393346	0%	10%	
TTN	chr2:179413687	14%	0%		TTN	chr2:179404199	13%	0%	
TTN	chr2:179414436	14%	0%		TTN	chr2:179413565	13%	0%	
TTN	chr2:179414817	14%	0%		TTN	chr2:179422215	13%	0%	
TTN	chr2:179415929	14%	0%		TTN	chr2:179425264	0%	10%	
TTN	chr2:179416935	0%	10%		TTN	chr2:179427497	13%	0%	
TTN	chr2:179417152	0%	10%		TTN	chr2:179429150	13%	0%	
TTN	chr2:179417452	14%	0%		TTN	chr2:179432641	13%	0%	
TTN	chr2:179419226	0%	10%		TTN	chr2:179434267	13%	0%	
TTN	chr2:179421596	14%	0%		TTN	chr2:179436754	13%	0%	
TTN	chr2:179421791	0%	10%	rs183013408	TTN	chr2:179441457	13%	0%	
TTN	chr2:179422214	14%	0%		TTN	chr2:179455560	13%	0%	
TTN	chr2:179424856	14%	0%		TTN	chr2:179458072	13%	0%	
TTN	chr2:179432185	14%	10%		TTN	chr2:179460461	13%	0%	
TTN	chr2:179433832	14%	0%		TTN	chr2:179462635	0%	10%	
TTN	chr2:179434798	14%	0%		TTN	chr2:179474277	0%	10%	
TTN	chr2:179435903	0%	10%		TTN	chr2:179481277	13%	0%	
TTN	chr2:179437494	14%	0%		TTN	chr2:179482201	13%	0%	
TTN	chr2:179439611	14%	0%		TTN	chr2:179485631	0%	10%	
TTN	chr2:179440635	14%	0%		TTN	chrX:179494968	0%	10%	rs192766485
TTN	chr2:179440995	14%	0%		TTN	chr2:179500768	0%	10%	
TTN	chr2:179444512	14%	0%		TTN	chr2:179553427	13%	0%	
TTN	chr2:179444789	14%	0%		TTN	chr2:179566946	0%	10%	
TTN	chr2:179447898	0%	10%		TTN	chr2:179570047	0%	10%	
TTN	chr2:179449116	0%	10%		TTN	chr2:179572323	0%	10%	
TTN	chr2:179453343	14%	10%	rs191549948	TTN	chr2:179582796	13%	0%	
TTN	chr2:179455677	0%	10%		TTN	chr2:179594134	13%	0%	
TTN	chr2:179456083	14%	0%		TTN	chr2:179596188	13%	0%	
TTN	chr2:179458118	14%	0%		TTN	chr2:179599069	13%	0%	
TTN	chr2:179458151	14%	0%		TTN	chr2:179599521	13%	0%	
TTN	chr2:179464371	14%	0%		TTN	chr2:179600264	13%	0%	
TTN	chr2:179466511	14%	0%		TTN	chr2:179604611	13%	0%	
TTN	chr2:179469558	14%	0%		TTN	chr2:179605815	0%	10%	rs201888760
TTN	chr2:179473055	14%	0%		TTN	chr2:179614489	13%	0%	
TTN	chr2:179473455	0%	10%		TTN	chr2:179614883	13%	0%	
TTN	chr2:179476243	0%	10%		TTN	chr2:179615326	13%	0%	rs142848087
TTN	chr2:179476610	14%	0%		TTN	chr2:179632544	13%	0%	
TTN	chr2:179483493	14%	0%		TTN	chr2:179634421	25%	40%	rs200875815
TTN	chr2:179483524	14%	0%		TTN	chr2:179636009	0%	10%	
TTN	chr2:179486004	14%	0%		TTN	chr2:179650587	0%	10%	rs199507913
TTN	chr2:179501253	14%	0%		TTN	chr2:179650627	13%	0%	
TTN	chr2:179504807	14%	0%		TTN	chr2:179664619	13%	0%	
TTN	chr2:179516237	14%	0%		RBMX	chrX:135956408	75%	70%	rs76876438
TTN	chr2:179535890	14%	0%		RBMX	chrX:135956462	50%	50%	rs74463481
TTN	chr2:179549399	14%	0%		RBMX	chrX:135956506	50%	40%	rs77794331
TTN	chr2:179571461	14%	0%		RBMX	chrX:135956573	50%	10%	
TTN	chr2:179575569	14%	0%		RBMX	chrX:135957672	0%	10%	rs139356075
TTN	chr2:179583286	14%	0%		RBMX	chrX:135957690	0%	10%	rs150541875
TTN	chr2:179587640	14%	0%		RBMX	chrX:135957700	0%	10%	rs139954333
TTN	chr2:179590307	14%	0%		RBMX	chrX:135957716	0%	10%	rs142284545
TTN	chr2:179594158	14%	0%		RBMX	chrX:135958704	0%	10%	rs112089728
TTN	chr2:179594648	14%	0%		RBMX	chrX:135958730	0%	10%	rs78702689
TTN	chr2:179594867	14%	0%		RBMX	chrX:135960119	13%	10%	rs76812369

Variant list for the top three variant AML-candidate genes (see Figure2, Panel B) - page 2 of 2									
AML Infants					ALL Infants				
Gene	Position	Infant Frequency	Mother Frequency	dbSNP ID	Gene	Position	Infant Frequency	Mother Frequency	dbSNP ID
TTN	chr2:179594933	14%	0%		RBMX	chrX:135960147	50%	20%	
TTN	chr2:179595693	0%	10%		RBMX	chrX:135961560	13%	10%	rs80321628
TTN	chr2:179599124	14%	0%		RBMX	chrX:135961585	0%	10%	
TTN	chr2:179600360	14%	0%		MLL3	chr7:151842305	13%	0%	
TTN	chr2:179605985	14%	0%		MLL3	chr7:151860230	0%	10%	rs142835638
TTN	chr2:179606240	14%	0%		MLL3	chr7:151873435	13%	0%	
TTN	chr2:179610430	14%	0%		MLL3	chr7:151902197	0%	10%	rs138119145
TTN	chr2:179610827	14%	0%		MLL3	chr7:151927016	0%	10%	rs141049734
TTN	chr2:179610922	14%	0%		MLL3	chr7:151927025	13%	0%	rs183684706
TTN	chr2:179610943	14%	0%		MLL3	chr7:151932945	25%	10%	rs199504848
TTN	chr2:179610988	14%	0%		MLL3	chr7:151945072	75%	40%	rs150073007
TTN	chr2:179610989	14%	0%		MLL3	chr7:151945228	0%	10%	rs200184971
TTN	chr2:179611336	0%	10%		MLL3	chr7:151960181	13%	0%	
TTN	chr2:179612343	14%	0%		MLL3	chr7:151962265	0%	30%	rs201834857
TTN	chr2:179613163	14%	0%		MLL3	chr7:151970877	13%	0%	rs138627563
TTN	chr2:179634421	14%	50%	rs200875815					
TTN	chr2:179640696	14%	0%						
TTN	chr2:179642589	0%	10%						
TTN	chr2:179659744	0%	10%						
TTN	chr2:179669360	14%	0%						
RBMX	chrX:135956408	43%	60%	rs76876438					
RBMX	chrX:135956462	29%	60%	rs74463481					
RBMX	chrX:135956506	29%	40%	rs77794331					
RBMX	chrX:135956573	29%	30%						
RBMX	chrX:135960147	14%	0%						
RBMX	chrX:135961560	14%	0%	rs80321628					
RBMX	chrX:135961585	0%	10%						
MLL3	chr7:151841869	14%	0%						
MLL3	chr7:151875022	14%	0%						
MLL3	chr7:151884389	14%	0%						
MLL3	chr7:151891103	14%	0%						
MLL3	chr7:151902304	14%	0%						
MLL3	chr7:151919134	14%	0%						
MLL3	chr7:151927025	43%	20%	rs183684706					
MLL3	chr7:151932945	29%	0%	rs199504848					
MLL3	chr7:151945072	86%	30%	rs150073007					
MLL3	chr7:151945225	0%	10%	rs202098135					
MLL3	chr7:151949795	14%	0%						
MLL3	chr7:151962134	0%	10%	rs146238849					
MLL3	chr7:151962168	0%	10%	rs138908625					
MLL3	chr7:151962265	0%	10%	rs201834857					
MLL3	chr7:151970859	14%	0%	rs149992209					
MLL3	chr7:152012416	14%	0%						
MLL3	chr7:152027753	14%	0%						
MLL3	chr7:152055740	14%	0%						

Supplementary Table 4. Individual variant listings for each rare, non-synonymous, predicted deleterious variant called variants meeting sequencing threshold parameters (see Methods) in the top three most commonly variable genes from the AML-candidate gene list (see Figure 2, Panel B). Each row lists an individual variant by gene, position (hg19), minor allele frequency across all infant exomes, minor allele frequency across all maternal exomes, and the dbSNP identification number (if applicable, blank means the variant is novel).

Variant list for the top three variant ALL-candidate genes (see Figure2, Panel A) - page 1 of 1									
AML Infants					ALL Infants				
Gene	Position	Infant Frequency	Mother Frequency	dbSNP ID	Gene	Position	Infant Frequency	Mother Frequency	dbSNP ID
MDN1	chr6:90377741	14%	0%		MDN1	chr6:90363929	0%	10%	
MDN1	chr6:90383180	14%	0%		MDN1	chr6:90363955	13%	0%	
MDN1	chr6:90383935	14%	0%		MDN1	chr6:90368355	13%	0%	rs115792683
MDN1	chr6:90388424	14%	0%		MDN1	chr6:90368471	13%	0%	
MDN1	chr6:90398446	14%	0%		MDN1	chr6:90368490	13%	0%	
MDN1	chr6:90405449	0%	10%		MDN1	chr6:90372565	13%	0%	
MDN1	chr6:90405586	0%	10%		MDN1	chr6:90385883	13%	0%	
MDN1	chr6:90420493	14%	0%		MDN1	chr6:90385922	13%	0%	
MDN1	chr6:90424435	0%	10%	rs150248107	MDN1	chr6:90396621	13%	0%	
MDN1	chr6:90434947	14%	0%		MDN1	chr6:90434940	13%	0%	
MDN1	chr6:90450026	0%	10%	rs114779526	MDN1	chr6:90448168	13%	0%	
MDN1	chr6:90459345	14%	0%		MDN1	chr6:90504494	13%	0%	
MDN1	chr6:90513126	14%	0%		SYNE1	chr6:152461288	13%	0%	
MDN1	chr6:90513189	0%	10%	rs143308656	SYNE1	chr6:152462353	13%	0%	
SYNE1	chr6:152443578	14%	0%		SYNE1	chr6:152462387	13%	0%	
SYNE1	chr6:152563445	14%	0%		SYNE1	chr6:152532645	0%	10%	
SYNE1	chr6:152570334	14%	0%	rs149272010	SYNE1	chr6:152542011	0%	10%	
SYNE1	chr6:152576099	0%	10%		SYNE1	chr6:152576794	13%	0%	
SYNE1	chr6:152577804	14%	0%		SYNE1	chr6:152642378	13%	0%	
SYNE1	chr6:152631566	14%	10%	rs145899734	SYNE1	chr6:152644741	0%	10%	
SYNE1	chr6:152642398	0%	10%	rs140850000	SYNE1	chr6:152658104	13%	0%	
SYNE1	chr6:152688393	14%	0%		SYNE1	chr6:152804248	0%	10%	
SYNE1	chr6:152720877	14%	0%	rs112061681	MLL2	chr12:49425964	0%	10%	rs200315963
SYNE1	chr6:152737745	14%	0%		MLL2	chr12:49426773	13%	0%	
SYNE1	chr6:152763314	14%	0%		MLL2	chr12:49428694	0%	10%	rs146044282
SYNE1	chr6:152771967	0%	10%	rs141464488	MLL2	chr12:49432416	0%	10%	
SYNE1	chr6:152777056	14%	0%		MLL2	chr12:49433599	0%	10%	rs147706410
SYNE1	chr6:152787119	14%	0%		MLL2	chr12:49434934	0%	10%	
MLL2	chr12:49418376	14%	0%		MLL2	chr12:49435457	0%	10%	
MLL2	chr12:49420207	0%	10%		MLL2	chr12:49441813	13%	0%	
MLL2	chr12:49421675	14%	0%						
MLL2	chr12:49425287	0%	10%						
MLL2	chr12:49426100	0%	10%						
MLL2	chr12:49436413	14%	0%						
MLL2	chr12:49443500	14%	0%						
MLL2	chr12:49444031	0%	10%						
MLL2	chr12:49444700	14%	0%						
MLL2	chr12:49445392	0%	10%	rs202076833					
MLL2	chr12:49445967	14%	0%						
MLL2	chr12:49447294	14%	0%						
MLL2	chr12:49448150	14%	0%						

Supplementary Table 5. Individual variant listings for each rare, non-synonymous, predicted deleterious variant called meeting sequencing threshold parameters (see Methods) in the top three most commonly variable genes from the ALL-candidate gene list (see Figure 2, Panel A). Each row lists an individual variant by gene, position (hg19), minor allele frequency across all infant exomes, minor allele frequency across all maternal exomes, and the dbSNP identification number (if applicable, blank means the variant is novel).
